# Co-electrodeposition of hard Ni-W/diamond nanocomposite coatings

**DOI:** 10.1038/srep22285

**Published:** 2016-02-29

**Authors:** Xinyu Zhang, Jiaqian Qin, Malay Kumar Das, Ruru Hao, Hua Zhong, Adisak Thueploy, Sarintorn Limpanart, Yuttanant Boonyongmaneerat, Mingzhen Ma, Riping Liu

**Affiliations:** 1 State Key Laboratory of Metastable Materials Science and Technology, Yanshan University, Qinhuangdao 066004, P.R. China; 2 Metallurgy and Materials Science Research Institute, Chulalongkorn University, Bangkok 10330, Thailand; 3 International Graduate Program of Nanoscience &Technology, Chulalongkorn University, Bangkok 10330, Thailand

## Abstract

Electroplated hard chrome coating is widely used as a wear resistant coating to prolong the life of mechanical components. However, the electroplating process generates hexavalent chromium ion which is known carcinogen. Hence, there is a major effort throughout the electroplating industry to replace hard chrome coating. Composite coating has been identified as suitable materials for replacement of hard chrome coating, while deposition coating prepared using traditional co-deposition techniques have relatively low particles content, but the content of particles incorporated into a coating may fundamentally affect its properties. In the present work, Ni-W/diamond composite coatings were prepared by sediment co-electrodeposition from Ni-W plating bath, containing suspended diamond particles. This study indicates that higher diamond contents could be successfully co-deposited and uniformly distributed in the Ni-W alloy matrix. The maximum hardness of Ni-W/diamond composite coatings is found to be 2249 ± 23 Hv due to the highest diamond content of 64 wt.%. The hardness could be further enhanced up to 2647 ± 25 Hv with heat treatment at 873 K for 1 h in Ar gas, which is comparable to hard chrome coatings. Moreover, the addition of diamond particles could significantly enhance the wear resistance of the coatings.

Composite plating is a technique involving co-electrodeposition of inert particles with metal/alloy in order to promote the hardness, wear and corrosion properties of the coatings, which has a great application in the industries. The composite coatings are prepared by co-electrodeposition of second-phase particles into a metal/alloy matrix, which show excellent higher hardness, lubricity, and wear properties^1^,^2^,^3^,^4^,^5^. The content of particles incorporated into a coating may fundamentally affect its properties. While deposition coatings fabricated using traditional co-deposition techniques have relatively low particles content^6^, the use of low-cost composite electroplating methods continues to expand and addresses the major challenge of achieving high levels of co-deposited particles. On the other hand, the hardness of composite coatings are controlled not only by the content of incorporated particles, but also by the hardness of the matrix^7^. Ogihara *et al*.^7^ reported that the hardness of Ni-B/diamond coatings was 1940 Hv. The hardness of the composite coating increased from 1940 Hv to 2494 Hv by heat treatment at 673 K for 1 h in air, comparable to hard chrome coatings and hard coatings prepared by dry processes. For example, the hardness of hard chrome coating is 850–1100 Hv^8^, and the hardness of TiN coatings deposited by dry process sputtering or supersonic plasma spraying is 2000–2700 Hv. Furthermore, Ogihara *et al*.^9^ also prepared the hard Ni-B/diamond composite coatings (micro-hardness 1248 Hv) by one-step electrodeposition. The hardness of composite coatings was further enhanced up to 2310 Hv by heat treatments, comparable to electroplated hard chrome coating, TiN coatings prepared by dry process and Ni-B/diamond composite coatings prepared by two-step wet process.

Electrodeposited Ni-W alloys were recently developed as a candidate to replace the environmentally hazardous hexavalent hard chromium coatings. The hardness of Ni-W can reach up to 700 Hv by controlling their grain size into the nanocrystalline regime^10^. Furthermore, the hardness of Ni-W coatings can be enhanced from 700 Hv to 1050 Hv by heat treatment^11^. According to these results, it is suggested that Ni-W alloys could be good candidate of matrix for diamond composite coatings. Hou *et al*.^12^ and Wang *et al*.^12^ successfully prepared the Ni-W/diamond composite coatings by electrodeposition. The microhardness reached a maximum (1205 Hv) after annealing at 600 °C due to the precipitation of the Ni_4_W phase. Zhang *et al*.^13^ also prepared Ni-W/diamond composite coatings using pulse current electrodeposition. The maximum hardness of as-deposited coatings was 988 Hv. The hardness of Ni-W/diamond, however, cannot be compared to the Ni-B/diamond composite coatings, which could be caused by the relatively low diamond particles content in the deposits. Consequently, in the present study, we report a simple one-step sediment co-electrodeposition (SCD) process for preparing hard Ni-W/diamond composite coatings.

## Results and Discussions

### Synthesis of the Ni-W/diamond coatings

**Figure 1:**
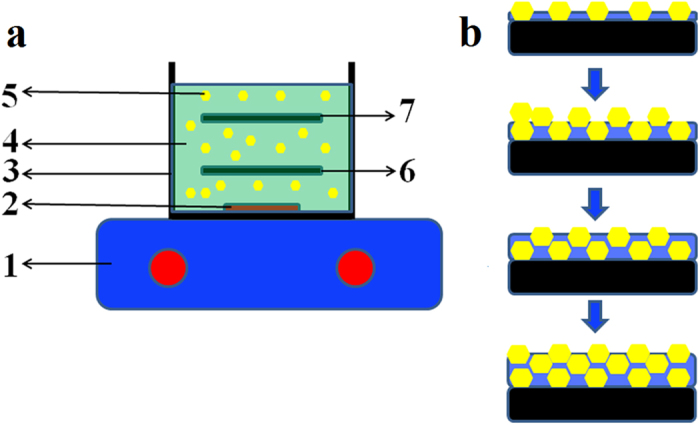
Schematic representation of the Ni-W-diamond composite coatings formation process through SCD method. (**a**) The deposition setup, 1-magnatic hot plate, 2- stirrer, 3-beaker, 4-electrolyte, 5-diamond particles, 6-steel cathode, 7-anode. (**b**) The schematic representation of composite coatings formation process through SCD method.

Ni-W and Ni-W/diamond coatings have been conventionally prepared by the electrodeposition method^12^,^14^. However, it is difficult to co-deposit diamond particles into the formed matrix with the conventional electrodeposition method. Therefore, the diamond content in Ni-W/diamond composite coatings prepared using the conventional electrodeposition is low, so that the hardness of diamond particles would make only a minor contribution to the hardness of the obtained coatings. We have previously demonstrated the preparation of composite coatings using SCD method^15^. Figure 1(a) shows the setup of SCD. Figure 1(b) shows the schematic representation of the Ni-W-diamond composite coatings formation process through SCD method. In the typical process, diamond particles will be suspended in the electrolyte solution during the deposition. Therefore, diamond particles could be easily co-electrodeposited with metal ions on the substrate because of the gravity of diamond particles. This SCD fabrication process can significantly improve the content of diamond particles in the deposits. In this study, the co-electrodeposition of Ni-W and diamond was conducted in a 200 mL plating bath with aqueous bath chemistry. The bath composite and plating conditions are listed in Table 1. Analytical reagents and deionized water were used to prepare the plating solution. The diamond particles of mean size of 0.8 and 3 μm were chosen to be co-deposited with nickel in the present experiments. Carbon steel was employed as a cathode. Prior to plating, steel substrates were successively washed in soap, rinsed in NaOH, HCl, and distilled water, and activated in 14%HCl. The steel substrates were masked with insulating tape with 4 cm^2^ of exposed area left. The steel substrate and Pt coated-Fe mesh plate with a distance of 35 mm in between were horizontally immersed into 200 ml of the electrodeposition baths. Electrodeposition was carried out under constant current density (0.05, 0.1, 0.15, and 0.2 A cm^−2^) and Ni-W/diamond composite coatings were successfully electrodeposited on the steel plates.

### Microstructure of the Ni-W/diamond coatings

**Figure 2:**
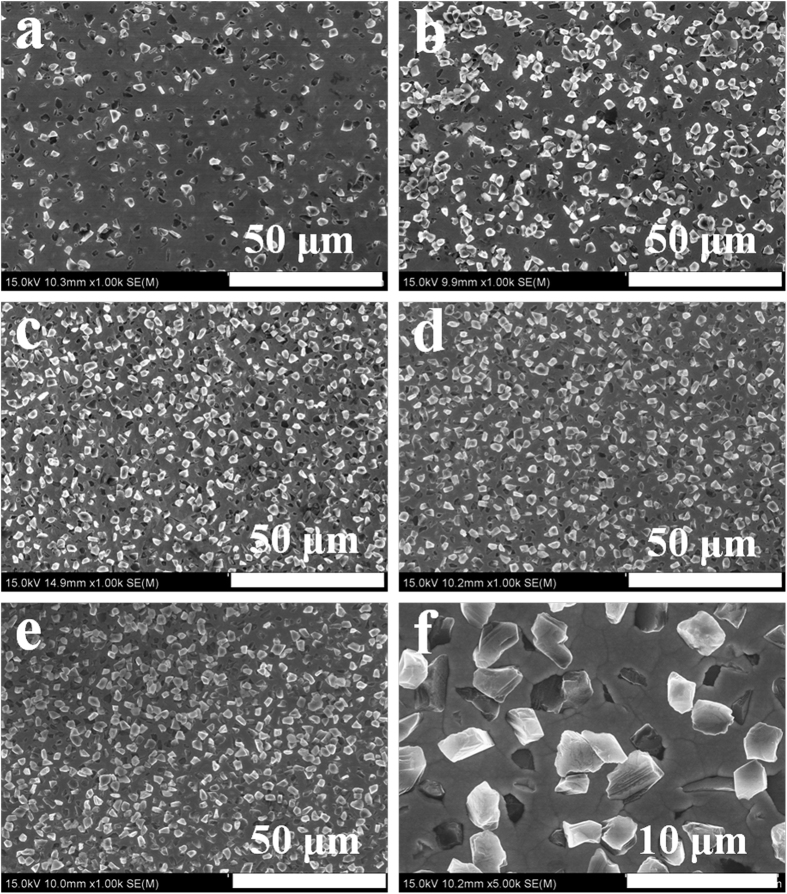
SEM images of Ni-W/diamond composite coatings. The coatings prepared under deposition current density of 0.1 A/cm^2^, bath temperature of 75 °C, and different diamond concentration in solution, (**a**) 1 g L^−1^, (**b**) 2 g L^−1^, (**c**) 5 g L^−1^, (**d**) 10 g L^−1^, (**e**) 20 g L^−1^. (**f**) the enlarged area of (**d**).

The scanning electron microscopy (SEM) images of Ni-W/diamond composite coatings are shown as Fig. 2, where Fig. 2(a–e) show the surface morphology of a Ni-W/diamond coatings fabricated via sediment co-deposition technique with current density of 0.1 A cm^−2^, bath temperature 75 °C, and different diamond concentration in bath, 1 g L^−1^, 3 g L^−1^, 5 g L^−1^, 10 g L^−1^, and 20 g L^−1^, respectively. The particles in Fig. 2 represent the co-deposited diamond in Ni-W matrix. It can be seen that the diamond particles are embedded into the Ni-W matrix, and they are uniformly and finely present, indicating that the diamond particles were co-deposited into the Ni-W matrix one by one. This can be seen clearly in Fig. 2(f), which is the enlarged area of Fig. 2(d). Furthermore, it can be seen that more diamond particles were co-deposited in the matrix presented in Fig. 2(c–e) than in those shown in Fig. 2(a,b). These SEM micrographs indicate that the uniform distribution of diamond particles throughout the deposits, and the diamond content in deposits increased with the diamond concentration in bath from 1 to 5 g L^−1^, while above 5 g L^−1^ the diamond content in deposits kept almost constant.

**Figure 3:**
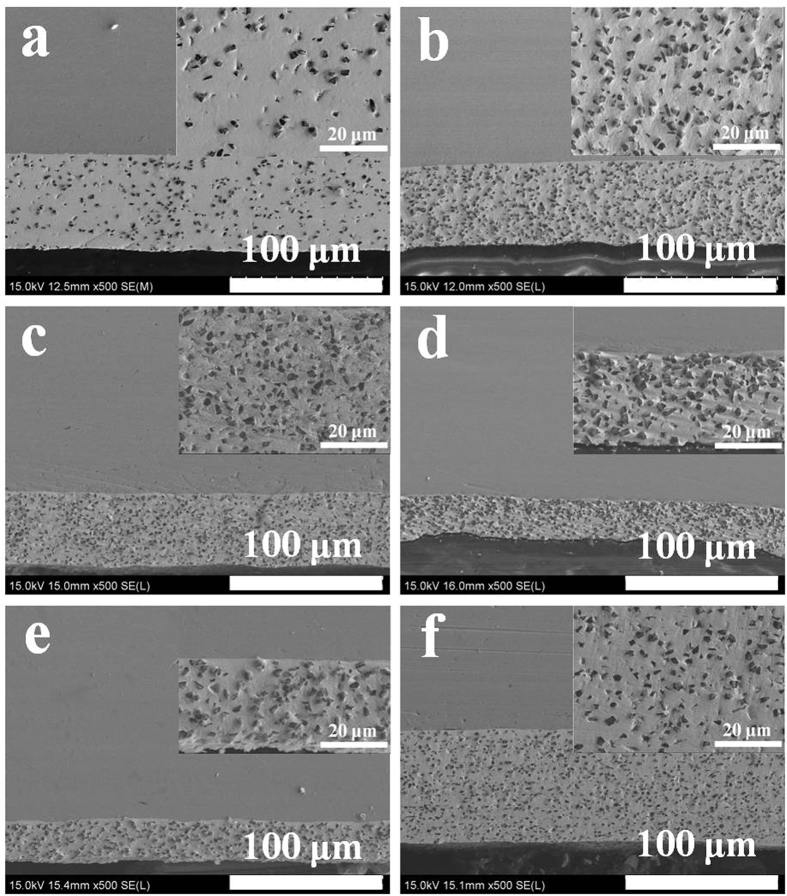
SEM image for cross-section of Ni-W/diamond composite coating under bath temperature of 75 °C. (**a**) diamond concentration of 1 g L^−1^ and current density of 0.1 A cm^−2^, (**b**) diamond concentration of 5 g L^−1^ and current density of 0.1 A cm^−2^, (**c**) diamond concentration of 10 g L^−1^ and current density of 0.1 A cm^−2^, (**d**) diamond concentration of 20 g L^−1^ and current density of 0.1 A cm^−2^, (**e**) current density of 0.05 A cm^−2^ and diamond concentration of 5 g L^−1^. (**f**) current density of 0.2 A cm^−2^ and diamond concentration of 5 g L^−1^.

Cross-sectional SEM images of Ni-W/diamond coatings prepared by the SCD method are also shown in Fig. 3. Figure 3(a–d) present the cross-section of a Ni-W/diamond coatings fabricated via SCD technique with current density of 0.1 A cm^−2^, bath temperature 75 °C, and different diamond concentration in bath, 1 g L^−1^, 5 g L^−1^, 10 g L^−1^, and 20 g L^−1^, respectively. The results reveal that the thickness of coatings is strongly affected by the diamond concentration in bath. As the diamond concentration is increased, the thickness of coatings decreases. The thickness of coatings for different current density (0.05 A cm^−2^, 0.1 A cm^−2^, and 0.2 A cm^−2^) was also examined, as shown in Fig. 3(b,e,f), respectively. It can be seen that the thickness increases with the current density. The thickness of the coating is *ca*. 25–70 μm with different deposition conditions. The inserted images show the enlarged cross section SEM images, which suggest that the coating is composed of dense diamond particles with diameters of *ca*. 2–4 μm. The results confirm that diamond particles are distributed uniformly in the Ni-W matrix, as expected.

**Figure 4:**
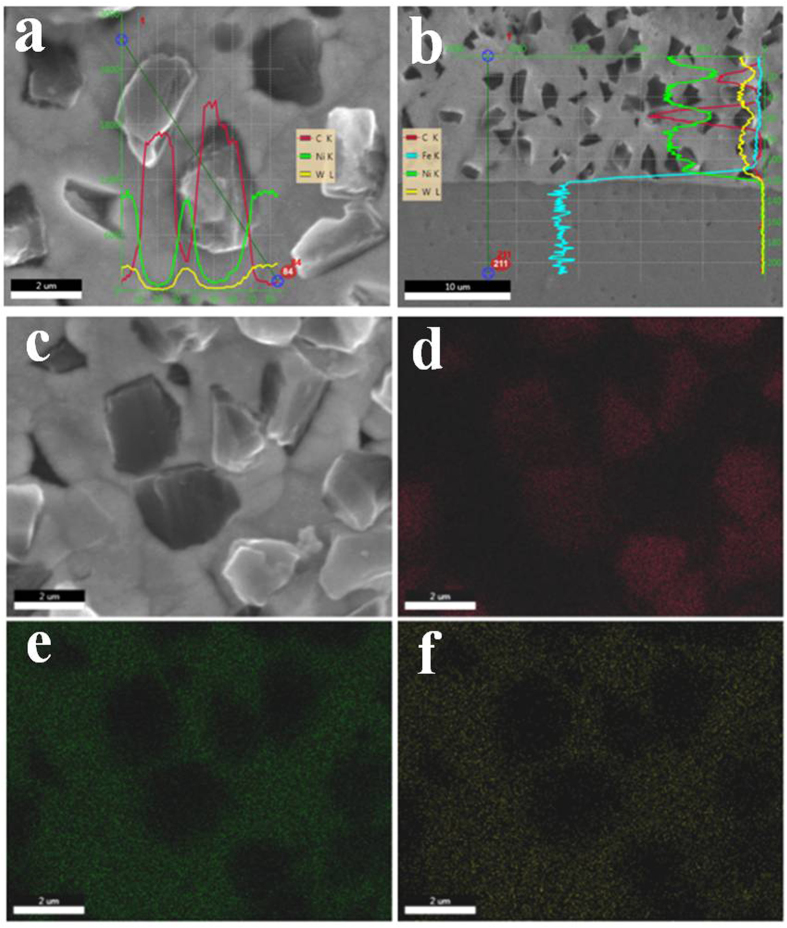
EDS analysis. (**a**) line EDS through diamond particles and Ni-W matrix on surface, (**b**) line EDS though diamond particles and Ni-W matrix on cross-section, (**c**) SEM image area of elemental mapping, (**d**) C element mapping, (**e**) Ni element mapping, (**f**) W element mapping.

The chemical composition of the as-deposited composite coatings was examined by energy dispersive X-ray spectroscopy (EDS) analysis. The W content is ~41–45 wt.%, as shown in Table 2. To determine the interface of chemical bonding and composition distribution between diamond particles and Ni-W matrix, the line EDS and X-ray elemental mapping were also carried out, as presented in Fig. 4. Figure 4(a,b) show the EDS analysis for the surface and cross section, respectively. Here, line scan EDS through the diamond particles zone and Ni-W matrix was performed. The results imply that Ni-W matrix showed the Ni and W spectrum and diamond particles zone that only presented the carbon spectrum. Thus, it indicates that diamond particles embedded into the Ni-W matrix by mechanical interlocking with Ni-W electroplating. Furthermore, the X-ray elemental mappings of Ni, W, and C along with the SEM image of the Ni-W/diamond composite coatings are shown in Fig. 4(d–f). The X-ray elemental mapping of C further confirms the uniform distribution of the diamond particles in the Ni-W matrix.

**Figure 5:**
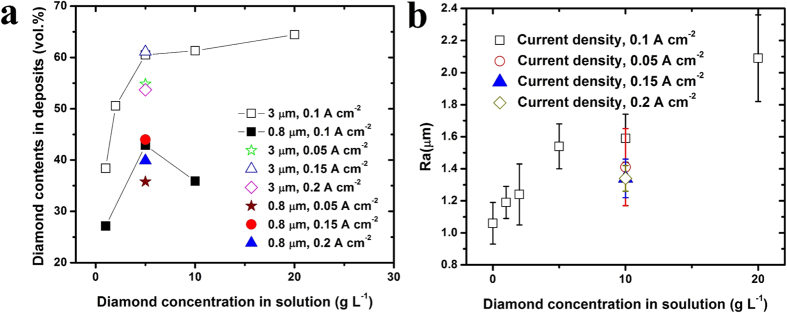
Diamond contents and surface roughness of Ni-W/diamond composite coatings. (**a**) The diamond particles content in deposits increased quickly with increasing in diamond concentration from 0 to 5 g L^−1^, while above 5 g L^−1^, the diamond content kept constant, (**b**) Surface roughness (Ra) increases with diamond concentration increasing, while the Ra is almost same for the composite coatings deposited at different current density.

The diamond contents in the composite coatings was evaluated using the gravimetrically method. Figure 5(a) shows the diamond contents in the coatings prepared with different diamond concentration, particles size, and current density. The results reveal that, as the diamond concentration in bath is increased from 0 to 5 g L^−1^, the diamond particles content in deposits increased quickly, while the diamond concentration in bath is above 5 g L^−1^, the diamond content in coatings kept almost constant. This could be caused by the blocking effect of the powder on the surface area available for plating^16^. Furthermore, the larger diamond particles embedded into the Ni-W matrix is better than that of smaller diamond particles (Fig. 5(a)). During the SCD process, particles are adsorbed onto the growing film surface in two successive steps and then are embedded within the electrodeposited metal matrix. Probably, diamond particles of 0.8 μm were too light to drop off on the substrate surface, resulting that the less of smaller diamond particles embedded into the metal matrix. In contrast, 3 μm of diamond particles were uniformly co-deposited into the Ni-W matrix, which is associated with the conditions that the diamond particles were dispersed uniformly in the bath and constantly dropped off and adsorbed on the cathode surface well.

The surface roughness of Ni-W and Ni-W/diamond coatings was examined by surface profilometer as shown in Fig. 5(b). The surface roughness (*Ra*) of Ni-W coatings is 1.06 μm. While the *Ra* of Ni-W/diamond (3 μm) coatings increases from 1.19 to 2.09 μm with the diamond concentration in bath from 1 to 20 g L^−1^. Moreover, as the diamond concentration in bath is 5 g L^−1^, the *Ra* of Ni-W/diamond coatings is 1.41, 1.54, 1.34, and 1.34 μm with current density of 0.05, 0.10, 0.15, and 0.20 A cm^−2^, respectively. Although *Ra* is 1-2 μm, the composite coatings have relatively flat surface.

**Figure 6:**
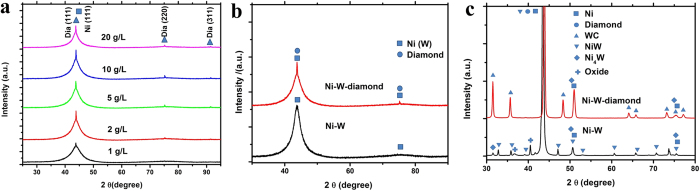
XRD analysis of the obtained samples. (**a**) XRD patterns of Ni-W/diamond composite coatings obtained at diamond concentration of 1, 2, 5, 10, 20 g L^−1^, in their as-plated condition, (**b**) XRD patterns of as-deposited Ni-W and Ni-W-diamond coatings, (**c**) XRD patterns of heat treatment of Ni-W and Ni-W/diamond coatings.

The X-ray diffraction (XRD) patterns of Ni-W/diamond composite coatings were presented as Fig. 6, where Fig. 6(a) shows the XRD of Ni-W/diamond composite coatings obtained at diamond concentration of 1, 2, 5, 10, 20 g L^−1^, in their as-plated condition. Three peaks occurred at 2theta angle of ~44°, ~75.3°, and ~91.5° which are indexed to diamond (JCPDS File No. 06-0675). And the broadened peaks at centre ~44° and ~76° could be matched with the Ni (JCPDS File No. 04-0850). This signifies the formation of solid solution of W in Ni and the peaks can be ascribed to (111) and (200) of the face-centered cubic (FCC)-Ni. Furthermore, the XRD patterns of Ni-W and Ni-W/diamond composite coatings with diamond concentration of 5 g L^−1^ at current density of 0.1 A cm^−2^ were shown in Fig. 6(b). It can be seen that the sharp diamond (111) peak appears in a XRD diffractogram of Ni-W/diamond composite coatings (Fig. 6(a,b)). XRD results could further demonstrate that the Ni-W/diamond composite coatings were successfully fabricated by using SCD method.

It has been reported that the properties of Ni-W coatings can be enhanced by heat treatment^11^,^17^. The heat treatment can change the amorphous structure into a crystalline alloy structure, such as Ni_4_W, NiW, as shown in Fig. 6(c), which have higher hardness than the amorphous Ni-W coatings. Wang *et al*.^12^ reported that heat treatment can improve the hardness of Ni-W/diamond, which can reach up to 1205 Hv from 800 Hv (as deposited). In the present work, heat treatment was also performed under 873 K in Ar gas. Figure 6(c) shows the XRD patterns of Ni-W/diamond coating fabricated at current density of 0.10 A cm^−2^, with diamond size 3 μm and 5 g L^−1^. The as-prepared coating has a broad diffraction peak at around 2θ = 30–55° (Fig. 6(a)), which indicates an amorphous structure. The sharp peaks occurred at 2theta angle of ~44° which is indexed to diamond (JCPDS File No. 06-0675). After heat treatment at 873 K for 1 h in Ar gas, the broad peak has disappeared and diffraction peaks corresponding to Ni, diamond, WC, Ni_4_W, and NiW appear^12^. It also implies that the growth and coarsening of grain are promoted with an increase in heat treatment temperature. The grain size of obtained coatings was calculated from the width of the Ni (111) peaks observed in the XRD patterns using Scherrer's equation. The grain sizes before and after heat treatment for Ni-W alloy and Ni-W/diamond composite coating are displayed in Table 2. We note that in the present alloy and composite coatings system W is a grain refining element, owing to its subtle tendency for grain boundary segregation^18^,^19^,^20^; accordingly, across the samples in Table 2, grain size is in agreement with the previous work^20^.

### Mechanical properties of Ni-W/diamond composite coatings

**Figure 7:**
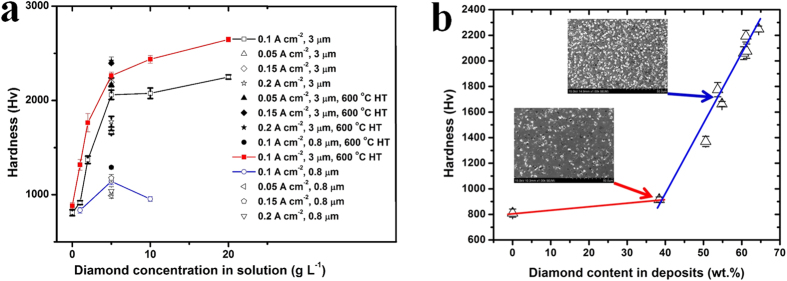
Hardness of coatings. (**a**) Hardness of the coatings with different deposition conditions and heat treatment, (**b**) hardness of the Ni-W alloys and Ni-W/diamond composite coatings presented as a function of the diamond content in coatings.

Figure 7 presents the hardness measurement results of Ni-W and Ni-W/diamond coatings. As is seen, without diamond additions, the electrodeposited Ni-W alloys fabricated with current density of 0.1 A cm^−2^, exhibit the hardness of 810 ± 32 Hv, which is in good agreement with the reported value^21^. The composite coatings prepared with diamond particles concentrations in bath of 1, 2, 5, 10, 20 g L^−1^, exhibit the hardness of 916 ± 20, 1370 ± 41, 2060 ± 52, 2076 ± 59, and 2249 ± 23 Hv, respectively. From Fig. 7(a), it can be seen that the incorporations of diamond can result in a marked improvement of hardness. The hardness increased with the concentration of diamond particles in the bath from 0 to 5 g L^−1^, while above 5 g L^−1^, the hardness did not cause significant differences. From the SEM (Fig. 2) and diamond content results (Fig. 5(a)), the diamond content in deposits increased quickly with increasing in the diamond concentration from 0 to 5 g L^−1^, while the diamond content was very stable above 5 g L^−1^. The results may be due to the blocking effect of the powder on the surface area available for plating^16^. Considering the mechanical properties of materials, the hardness of Ni-W/diamond composite coatings is controlled by three factors: co-deposition of diamond particles, the W content and grain size of metal matrix. According to the EDS and XRD results (Table 2), the W content (~40–44 wt.%) and grain size of metal matrix (~1.1 nm) did not have a significant change. Thus, the hardness of Ni-W/diamond composite coatings is mainly contributed by diamond content in deposits. In order to understand how the diamond content in deposits affects the hardness of coatings, it is therefore useful to represent the hardness of diamond-free and diamond incorporated Ni-W alloys as a function of the diamond content in the deposits, which was shown in Fig. 7(b). From this figure, it can be observed that the diamond content in deposits strongly influences the hardness. The hardness increased quickly with the diamond content. Figure 7(a) also shows the effect of diamond size and current density on the hardness of Ni-W/diamond composite coatings. The hardness of Ni-W/diamond (3 μm) composite coatings is about 2.5 times higher than those of Ni-W/diamond (0.8 μm) coatings, which follows the same trend as the diamond contents in the deposits (Fig. 5(a)). The diamond content of Ni-W/diamond (0.8 μm) coatings were lower than those of Ni-W/diamond (3 μm) coatings. We consider that sedimentation contributes to these results. As sedimentation has a strong influence on large particles, therefore, the larger particles could be easily dropped off and co-deposited into the Ni-W alloy, which results in a reduction of the diamond content at diamond particles of 0.8 μm. With the current density increasing, the hardness of Ni-W/diamond increased from 1663 ± 19 to 2193 ± 46 Hv, while the hardness slightly decreased above 0.15 A cm^−2^. A similar tendency was also observed in another electrodeposition system (Ni-B/diamond)^22^. To the best of our knowledge, the Ni-W/diamond coating is the hardest among coatings fabricated by wet chemical process. Moreover, the hardness of Ni-W/diamond coatings is higher than that of hard chrome coating (850–1100 Hv)^8^ and Ni-B/diamond coating (1940 Hv, load 50 gf)^7^,^9^ , which is even in the same range as the hard coatings prepared by dry process (e.g, TiN, 2000–2700 Hv).

The hardness with different deposition conditions after heat treatment was also examined, as shown in Fig. 7a. The results imply that the hardness of the Ni-W/diamond coatings increased from 2060 ± 52, 2076 ± 59, and 2249 ± 23 Hv to 2263 ± 37, 2437 ± 41, 2647 ± 25 Hv with diamond concentration 5, 10, 20 g L^−1^, respectively, with heat treatment at 873 K for 1 h in Ar gas. In order to examine the structural change of Ni-W/diamond coatings during heat treatment, XRD patterns were measured, as shown in Fig. 6(c). Heat treatment of the coating resulted in the crystal growth and the formation of WC hard phase and Ni_4_W, NiW alloy. Without diamond incorporation, oxide has be detected according to the XRD results, which agreed with the previous work^17^. However, the accurate phase identification could not be made but Ni-O, W-O, or Ni-W-O phases could be possible. While XRD results reveal no oxide was formed during the high temperature treatment for Ni-W/diamond composite coatings (Fig. 6(c)). According to the present results, the formation of WC hard phase in the Ni-W matrix and the precipitate of NiW and Ni_4_W alloy caused the hardening of the Ni-W/diamond coating.

**Figure 8:**
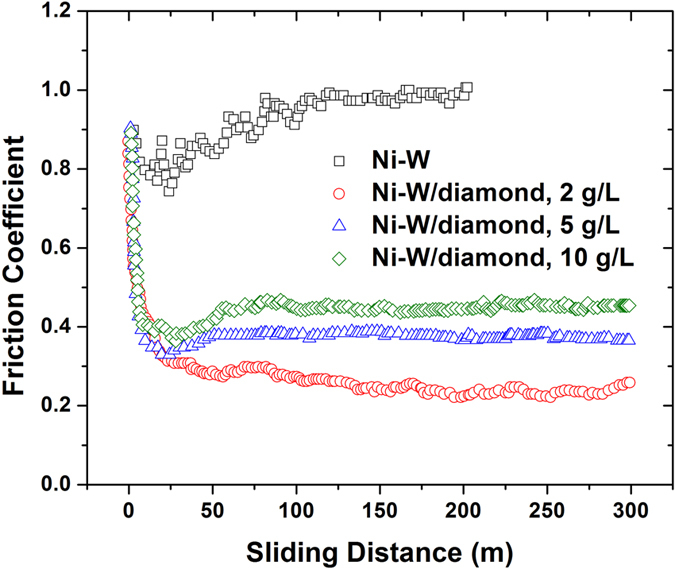
The friction coefficient during wear testing. With diamond incorporation, the fiction coefficient decreases.

The performance of many products and engineering components depends critically on tribological properties of surfaces such as wear and friction. Here the wear testing was also performed by using reciprocating-sliding tribometer for Ni-W (prepared at current density of 0.1 A/cm^2^) and Ni-W/diamond composite coatings (deposited at current density of 0.1 A/cm^2^ and diamond concentration of 2, 5, and 10 g/L). Figure 8 show the corresponding coefficient of friction (CoF) data for Ni-W and Ni-W/diamond composite coatings, respectively. For Ni-W coating the friction coefficient is always larger than those of Ni-W/diamond composite coatings. During the wear testing, if the maximum friction force is larger than 10 N, i.e. the CoF is larger than 1 in present case with the load of 10 N, the testing will be stopped by the protection function of the equipment. For Ni-W coatings, one test stopped when the sliding distance reached 42 m and it stopped at 200 m again at another test because the CoF became larger than 1. For Ni-W/diamond composite coatings, however, the CoF was kept almost constant ~0.2–0.45 for sliding distance of 300 m or even longer. For example, the friction coefficient of obtained coatings prepared at diamond concentration of 5 g/L can keep ~0.4 until the sliding distance approaching up to 600 m. The CoF reveals that the addition of diamond could significantly reduce the friction coefficient. Observing the wear tracks generated on treated samples can provide information on the wear performance and mechanics of reciprocating ball-on-plate sliding, of particular interest is the changes induced by addition of the diamond.

From the Fig. 8, it can be seen that the friction coefficient decreases sharply during running in and then stabilizes to steady-state. During the wear testing process, it takes place as the two surfaces are moving in relation to each other, and both physical and chemical changes occur. As a function of time the wear process changes in both geometry and the material composition. For the Ni-W/diamond composite coatings, in the initial phase of wear process, the ball will abrasive both Ni-W matrix and diamond particles, resulting the higher friction coefficient. Then, the diamond particles could be exposed and applied as micro-cutter. Despite being hard, diamond particles are known to have low friction coefficient. Therefore, the friction coefficient decreases and keeps stable in all obtained composite coatings. The reduction of friction coefficients can be explained by the formation of low-shear micro-points on the coating or perhaps only on the asperity tips of the coatings^23^. While the Ni-W coatings, the friction coefficient slightly increases and reaches up to 1 at about 40 m (one test) and 200 m (another test) sliding distance.

**Figure 9:**
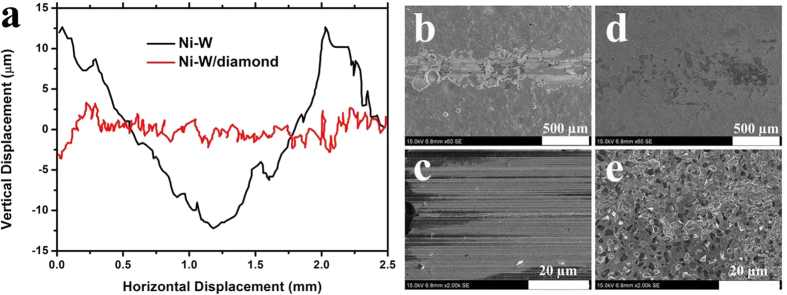
Worn surface observation. (**a**) Wear track profilometry scans for Ni-W coatings, and Ni-W/diamond composite coatings. (**b**) general SEM view of wear track of Ni-W coatings, (**c**) inside of the wear track of Ni-W coatings, (**d**) general SEM view of wear track of Ni-W/diamond composite coatings, (**e**) inside of the wear track of Ni-W/diamond composite coatings.

Figure 9(a) presents the 2D surface profilometry of wear scars. The wear scars can clearly demonstrate that the Ni-W/diamond composite coatings exhibit better wear resistance. From the 2D wear scars, the wear volume could be estimated for Ni-W coatings (0.55 mm^3^). However, the measurement of wear volume for Ni-W/diamond composite coatings is difficult because of the very less of removed coatings.

SEM was also performed to study the inside wear tracks of the Ni-W (sliding distance 42 m) and Ni-W/diamond composite coatings prepared with diamond concentration of 5 g/L (sliding distance 600 m), as shown in Fig. 9(b–e). From the Fig. 9(b,c) can be observed that the wear track of Ni-W coatings is clear and the smeared appearance of the surface is typical of material spalled, lots of scratching, as well as extensive plastic deformation. The morphology of worn surface of the Ni-W/diamond composite coatings is shown in Fig. 9(d,e). It can be observed that the wear track is not clear. No any diamond particles were peeled off during the wear test from the magnified SEM worn surface of Ni-W/diamond composite coatings (Fig. 9(e)) owing to the strong adherence between diamond particles and Ni-W matrix.

**Figure 10:**
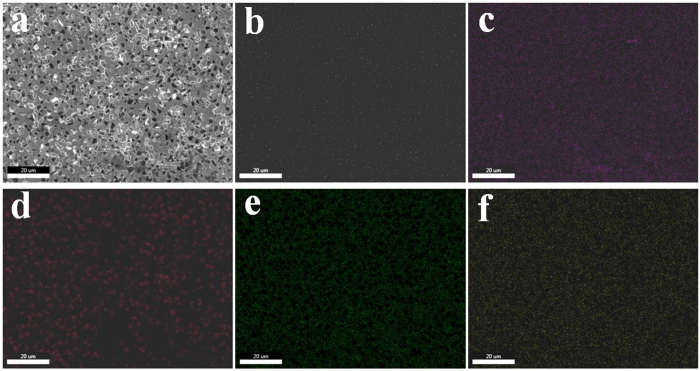
Elemental mapping of worn surface. (**a**) SEM image, (**b**) N element mapping, (**c**) Si element mapping, (**d**) C element mapping, (**e**) Ni element mapping, (**f**) W element mapping.

Due to the high hardness of the coatings deposited, balls used for testing were also worn. The SEM wear scar morphology has been attributed in part to adhesion of ball materials to the wear worn surface. This is also confirmed by the elemental mapping, as shown in Fig. 10, which indicates that elemental map showing the distribution of N, Si, C, Ni, W elements in the worn surface of Ni-W/diamond composite coatings. It can be seen that the concentrated position of red spot contain a higher proportion and uniformly of C elements. It shows diamond particles have extensive distribution in the worn surface of Ni-W/diamond composite coatings. The reinforced diamond particles have reduced the direct contact between the Ni-W matrix and the counter ball during the wear test that also reduced the friction coefficient and resulted in the higher wear resistance. Figure 10(b,c) show that the N and Si elements uniformly distribute on the worn surface, it suggests that the hardest diamond particles could easily remove the ball materials during the wear testing, and the removed ball materials adhere onto the worn surface.

In summary, sediment co-electrodeposition was performed to prepare hard Ni-W/diamond composite coatings. These coatings exhibited extreme high hardness and superior wear resistance. The hardness of the composite coatings was increased by increasing the concentration and size of diamond particles in present study because diamond particles with higher concentration and larger sized tend to co-deposit into Ni-W matrix more easily. Furthermore, the hardness was improved by heat treatment of the coatings. The hardest Ni-W/diamond composite coating in the present work was even higher than that of Ni-B/diamond coatings prepared by wet process and comparable to hard coatings prepared by dry processes.

## Methods

### Chemicals

Nickel(II) sulfate hexahydrate NiSO_4_·6H_2_O(Carlo), Sodium tungstate dihydrate Na_2_WO_4_·2H_2_O(Carlo), Tri-sodium citrate dihydrate Na_3_C_6_H_5_O_7_·2H_2_O(Carlo), Ammonium chloride NH_4_Cl(Carlo), Sodium bromide NaBr(Carlo) used in the present study were of analytical reagent grade. Diamond powder was from Huanghe Xuanfeng Co. Ltd., China. All aqueous solutions were prepared using double distilled water.

### Sample preparation

The co-electrodeposition of Ni-W and diamond was conducted in a 200 mL plating bath with aqueous bath chemistry. The bath composite and plating conditions are listed in Table 1. We have previously demonstrated the preparation of composite coatings using SCD method^15^. Figure 1(a) shows the setup of SCD. Figure 1(b) shows the schematic representation of the Ni-W-diamond composite coatings formation process through SCD method. Analytical reagents and deionized water were used to prepare the plating solution. The diamond particles of a mean particles size of 0.8 and 3 μm were chosen to co-deposit with nickel in the present experiments. The concentration of diamond particles in the bath are 1, 2, 5, 10, 20 g L^−1^. The bath temperature was maintained at 75 °C. The pH of the electrolyte was 8.9, and it was unaffected by the diamond additions. Carbon steel was employed as a cathode. Prior to plating, steel substrates were washed in soap, rinsed in NaOH, HCl, and distilled water, and activated in 14%HCl. The steel substrates were masked with insulated tape to leave 4 cm^2^ of exposed area.

The steel substrate and Pt coated-Fe mesh plate with a distance of 35 mm were horizontally immersed into 200 ml of the electrodeposition baths. Electrodeposition was carried out under constant current density (0.05, 0.1, 0.15, and 0.2 A cm^−2^). Ni-W/diamond composite coatings were electrodeposited on the steel plates. The coatings were washed with water and dried in air at room temperature. In addition, a Ni-W alloy coating was also obtained by SCD using the developed setup.

### Materials characterization

Prior to surface analysis, all coatings were washed in deionized water and ultrasonicated in acetone for 5 min. Optical microscope (OM) was used to determine the thickness of coating. Scanning electron microscopy (SEM, Hitachi, S4800) was performed to observe the surface of the coatings. The particle contents in the composite coatings was evaluated using the gravimetrically method. Deposits were stripped in nitric acid, which was filtered, and the mass of diamond powder in the deposit was estimated gravimetrically. The phases of the composite coatings were detected via X-ray diffraction using an X’Pert Pro diffractometer (Panalytical). The surface profile (Gauges, Ambs, US) was used to measure the surface roughness. Vickers microhardness for the surface of coatings was measured using a microhardness tester under an indentation load of 100 gf for 15 s at seven different locations of a specimen, and the average value of the five measurements (except the maximum and minimum values) is quoted as the hardness of the film.

Wear tests were performed using a CSM reciprocating-sliding tribometer, connected to a computer monitoring the dynamic coefficient of friction (in both sliding directions), relative humidity and temperature. Tests were performed by applying a normal load of 10 N to a stationary ball of diameter 6 mm. The ball materials used were Si_3_N_4_. The ball-on-plate machine was set to run at 100 mm/s with reciprocation amplitude of 10 mm and without lubrication. The tests performed at temperatures between 20 and 25 °C. Before each test, both the sample and the ball counterface were ultrasonically cleaned in acetone for 10 min, and dried by hot air. The anti-wear performance of the films was estimated from the weight loss of the specimens. After the wear tests, the morphology of each wear scar was observed by SEM. Also the SEM and EDS were used to obtain information regarding the morphology and chemical composition of the wear debris.

## Additional Information

**How to cite this article**: Zhang, X. *et al*. Co-electrodeposition of hard Ni-W/diamond nanocomposite coatings. *Sci. Rep*. **6**, 22285; doi: 10.1038/srep22285 (2016).

## Figures and Tables

**Table 1:** Bath compositions and deposition conditions.

Chemicals/Parameters	
NiSO_4_·6H_2_O	18 g L^−1^
Na_2_WO_4_·2H_2_O	53 g L^−1^
Na_3_C_6_H_5_O_7_·2H_2_O	168 g L^−1^
NH_4_Cl	31 g L^−1^
NaBr	18 g L^−1^
Diamond	0, 1, 2, 5, 10, 20 g L^−1^
Current density	0.05, 0.10, 0.15, and 0.20 A cm^−2^
Temperature	75 °C
pH	8.9

**Table 2:** Grain size and W content in the Ni-W/diamond nanocomposite coatings with different deposition conditions.

Deposition conditions			As-deposited		After heat treatment
Diamond size (μm)	Concentration (g L^−1^)	Current density (A dm^−2^)	Grain size (nm)	W content (wt.%)	Grain size (nm)
3	5	5	1.1	41.6	8.6
3	5	10	1.1	42.8	8.5
3	5	15	1.1	42.0	5.2
3	5	20	1.7	41.4	6.2
3	1	10	1.1	44.4	10.7
3	2	10	1.2	43.4	5.7
3	10	10	1.1	42.6	9.1
3	20	10	1.1	43.0	9.8
0.8	1	10	1.1	42.3	–
0.8	5	10	1.4	41.7	6.8
0.8	10	10	1.1	40.8	–
–	0	10	2.8	44.8	9.4
